# Acute Maternal Stress Disrupts Infant Regulation of the Autonomic Nervous System and Behavior: A CASP Study

**DOI:** 10.3389/fpsyt.2021.714664

**Published:** 2021-11-17

**Authors:** Isabelle Mueller, Nancy Snidman, Jennifer A. DiCorcia, Ed Tronick

**Affiliations:** ^1^Department of Medical Psychology and Medical Sociology, University Medical Center Göttingen, Georg-August University, Göttingen, Germany; ^2^Child Development Unit, Department of Psychology, University of Massachusetts Boston, Boston, MA, United States

**Keywords:** maternal stress, infant regulation, autonomic nervous system, infant stress reactivity, caretaker acute stress paradigm, still face paradigm

## Abstract

Exposure to maternal stress is assumed to influence infant health and development across the lifespan. The autonomic nervous system (ANS) is especially sensitive to the effects of the early caregiving environment and linked to predictors of later mental health. Understanding how exposure to maternal stress adversely affects the developing ANS could inform prevention. However, there is no agreed upon definition of maternal stress making its study difficult. Here we use the Caretaker Acute Stress Paradigm (CASP) to study the effects of maternal stress in an experimentally controlled laboratory setting. The CASP has 5 episodes, a natural play, followed by a caretaker stressor (or control) condition, another play, a classic still face episode, followed by another play. A total of 104 4-months-old infants and their mothers were randomly assigned to either the caretaker-stress or caretaker-control condition. Changes in behavior, heart rate (HR), and respiratory sinus arrhythmia (RSA) before and after the introduction of the stressor (or control condition) were recorded and compared. Infants in the maternal stress condition showed significantly more behavioral distress [*X*^2^ = (1, *N* = 104) = 4.662, *p* = 0.031]. Moreover, infants whose mothers were in the stress condition showed an significant increase in heart rate after the caretaker condition [*F*_(1, 102)_ = 9.81, *p* = 0.002]. Finally we observed a trend to faster RSA recovery in infants of the control condition [*F*_(1, 75)_ = 3.539, *p* = 0.064]. Results indicate that exposure to acute maternal stress affects infant regulation of the autonomic nervous system and behavior.

## Introduction

Early exposure to maternal stress influences health and development across the lifespan (1–6). Research in humans and animals suggests that exposure to maternal stress has long-term consequences on the offspring's stress reactivity (7), with a subsequently increased vulnerability for psychological disorders later in life (8–10). The autonomic nervous system (ANS) is especially sensitive to responding to the effects of the early caregiving environment (11–13) and crucial in the prediction of mental health (14, 15). However, research so far has paid little attention to the underlying mechanisms linking maternal stress to a dysregulation of the child's ANS.

### Development of Infant Regulatory Capacity

The early caregiver-infant interaction is a primary developmental context during the first year of life (16–18). While the infant's self-regulation capacity is not yet mature, the caregiver's sensitive and reliable co-regulation is crucial for the infant to cope with everyday stress. The caretaker-infant dyad usually cycles between matching with active co-regulation and dysregulated mismatching states (18, 19). These regulatory mismatches are not inherently harmful. Through reliable and repeated reparations of mismatched interactions, the infant learns that unwanted affective states and unbalanced interactions can be transformed into successful exchanges between both partners, leading to a better adaption to stress and probably future resilience (20, 21). However, if dysregulation becomes chronic and attempts to repair the interaction repeatedly fail, stress can become toxic for the child (22).

Models such as the Mutual Regulation Model (23) assume that maternal stress interferes with maternal caregiving capacity and leads to inconsistencies in the dyadic co-regulation (4, 20, 21, 24–26), which is hypothesized to be a crucial factor in the development of a child's stress response (23). Until self-regulatory capacities become more robust over the first year of life, the caregiver is a critical source of external regulation and has a key role in co-regulating the infant's emotion (16, 27). The maturation of self-regulation extends throughout childhood and the caregiver continues to serve a crucial co-regulatory function through the fourth to fifth year of life (28). Within the interaction, the caregiver's consistent and sensitive response helps the child organize its behavioral and physiological response to stress.

Regular and predictable regulatory scaffolding by the caretaker helps the infant learn how to regulate more effectively. Exposure to stress is hypothesized to lead to a depletion of resources (29, 30), resources that would otherwise be used for growth for the child or in the parent to co-regulate the dyadic interaction. When the exposure to stress becomes chronic, the diminishment of resources may result in long-lasting effects on the quality of the dyadic interaction and infant development (16, 17). Calming Cycle Theory [CCT (22)] further differentiates psychological co-regulation and physiological visceral-autonomic co-regulation. According to CCT, early shaping of visceral-autonomic co-regulation begins before birth through Pavlovian conditioning. The theory moves past attachment theory and connects how the emotional relationship between mother and infant is in part responsible for the development of the quality of the child's autonomic and behavioral regulation (31).

What unifies these theories is that all concur that a child's earliest experiences shape the development of self-regulatory capacities with long-lasting effects, for good or ill, on later mental health (17, 30, 32, 33). To date most research has focused on behavioral-affective regulation of infants in the presence of maternal stress. Recent studies on underlying biological mechanisms look at the infant's hormonal stress response, how sensitive caregiving buffers an increase of stress hormones and protects the developing brain from the potentially toxic, harmful effects (16, 16, 30, 34, 35). Even though the autonomic nervous system plays a critical role in reactivity to and regulation of stress and is sensitive to the infant's early experiences, little research, in particular experimental research has investigated its development in the context of maternal stress (36, 37).

### Role of the Autonomic Nervous System

The autonomic nervous system controls central and peripheral biophysiological responses to the environment. It goes through a period of rapid development from the last trimester of pregnancy well-into infancy, making it susceptible to environmental influences (38). The ANS has two systems that are engaged in ongoing regulation of cardiac function; the parasympathetic nervous system (PNS), which controls the body's physiological homeostasis at rest. The sympathetic nervous system (SNS) is more involved in activating the “fight or flight” response during a perceived threat. Activity of neurotransmitters that innervate the vagus nerve, the tenth cranial nerve connecting visceral organs through sensory fibers with the brain, is assumed to play a significant role in creating physiological resting state homeostasis as part of the PNS (39, 40). The PNS is involved in decreasing heartrate after stress exposure and plays a critical role in returning the body to its resting state. Therefore, vagus nerve activity is assumed to indicate PNS's neural regulation by decreasing arousal and returning the body to homeostasis after a confrontation with a stressor. Due to its role in the resting state recovery of the PNS, vagal tone has been linked to self-regulatory processes. However, vagus nerve activity itself is difficult to investigate directly and respiratory sinus arrhythmia (RSA), a measure of changes in HR linked to respiration, has emerged as a proxy (41). Heart rate accelerates with inhaling and decelerates with exhaling (42). RSA is assumed to increase with PNS activation and decrease with PNS withdrawal allowing HR to increase (37). Heart rate is controlled by both PNS and SNS, but can increase without observable changes in RSA, accordingly, RSA is commonly used as a measure of PNS activity (43). Additionally, the SNS has a slower frequency and is therefore hard to measure, especially in moving infants. Thus, the majority of research on infants ANS reactivity in the presence of a stressor focuses on average heart rate (HR), heart rate variability (HRV), and respiratory sinus arrhythmia (RSA).

The Still-Face (SF) paradigm has been widely used to study infant stress reactivity [for reviews, see (44, 45)]. A recent meta-analysis identified 33 peer-reviewed studies that investigate its impact on changes in measures related to the autonomic nervous system (37). While many of the reports differ in exact measurements or calculations, a majority describes very similar results. For instance, infant heart rate is often observed to increase from the natural play episode to the SF episode (46–49). Results are more equivocal for recovery of heart rate after the SF episode; some studies found no change in infant heart rate from SF stressor to the reunion (46, 49, 50), while others report a significant decrease in infant heart rate (47, 51). Gunning et al. (52) divided infants by the characteristic of neonatal irritability. They found that non-irritable infants showed a recovery of heart rate during the reunion after the SF while irritable infants did not.

Several studies also looked at the relation of maternal stress on infant stress reactivity. Enlow et al. (46) found that maternal trauma was linked to a less pronounced recovery of heart rate after the SF stressor. Stress during the prenatal period was associated with greater changes in infant RSA during the SF episode (38). Two studies investigating the effect of maternal anxiety found a reduced infant RSA during baseline (13, 53), and one study found higher infant RSA after the SF stressor for infants of mothers with elevated anxiety levels (54).

### The Current Study

However, most of these studies have two methodological flaws; they stress the infant, not the mother, and they vary widely in their definition of maternal stress. The term maternal stress has been used as an umbrella spanning various forms of adverse life conditions mothers may face, such as poverty, low SES, low social support, as well as mental health problems such as depression or anxiety (55–57). While all of these conditions may cause “stress,” there are large variations in how they may affect the caretaker's experience, physiology and everyday life. Moreover, it is difficult to differentiate which of them are closer to real toxic stress or daily challenges that many people face (55). Experimental studies that control for covariates usually stress the infant (e.g., still face experiment or cold pressure stress), studies that evaluate caretaker stress are often retrospective studies using correlational measures, making causal conclusions difficult (16, 58).

Aim of the present study is to evaluate the impact of standardized maternal stress in a controlled laboratory environment on infant behavioral and autonomic regulatory capacity. The Caretaker Acute Stress Paradigm (CASP) (59) allows studying the immediate effects of maternal stress, induced by infant cry vocalizations and distress images of infants on infant self-regulation, a clear and comparable definition of the construct (“maternal stress”). The cries of the caretaker condition were chosen as a stressor as infant cries have relevance to parenting, adding to the ecological validity of the paradigm. Previous research further indicates that infant cries produce a reliable stress response (60–62) and more distressing cries have been shown to recruit regions of the brain associated with arousal and attention (63, 64).

We hypothesized that infants of mothers in the caretaker-stress condition of the CASP would show decreased behavioral regulation and increased reactivity of the autonomic nervous system to the modeled caretaker-stress, compared to the infants of a caretaker-control group. Changes in behavior, heart rate (HR), and respiratory sinus arrhythmia (RSA) were recorded. Infant average HR and RSA before and after introducing a caretaker stressor or non-stressor control condition were compared to investigate how an acute experimental caretaker stressor may affect the infant's ability to self-regulate.

## Methods

### Participants

Participants were recruited at the maternity ward of a large Harvard Medical School-affiliated hospital. A hospital employee reviewed maternal and infant medical records for study inclusion and exclusion criteria (e.g., serious medical and/or mental maternal health issues). A recruiter visited the rooms of healthy full-term infants and their mothers to either talk with the mother about the study or to leave written material with contact information if the mother was unavailable. All potential participants were contacted 3 weeks before the infants were 4-months old.

A total of 104 4-months-old infants (+/−1 week) and their mothers participated and were randomly assigned to either the caretaker-stress or caretaker-control condition of the CASP before their arrival to the laboratory. The majority of mothers were white 54.7% (black: 26.4%, did not wish to answer: 9.4%). All infants were delivered full-term (37 weeks or greater) and were clinically healthy at birth as determined by pediatric examination, with no chronic medical conditions or time in the neonatal intensive care unit. Infants also were clinically healthy at the time of testing. Mothers were between 20- and 42 years of age at the time of birth, with no serious chronic health conditions and at least a high school education.

### Experimental Procedure

Participants came to the laboratory when the infant was 16-weeks old (+/−1 week). Informed consent was given, all questions were answered, and mothers signed the consent form. To collect cardiac data, seven electrodes (MindWare Technologies Ldt.) were placed on mother and infant. Infants were lying on a changing table in the waiting room, with one research assistant placing the electrodes on the infant. At the same time, a second research assistant placed the electrodes on the mother. Next, mother and infant were brought to the observation room where the infant was seated in a highchair while the mother sat on a chair facing the infant, close enough to touch and interact with the infant. The electrode wires from mother and infant were connected to MindWare, and the research assistant made sure the wires were tucked away so that the infant would not be able to reach them. Two wall-mounted video cameras were used to record mother and infant. Research assistants were able to monitor the study room and physiology from an adjacent control room. Physiology and video were initialized simultaneously through E-Prime® software to ensure exact timing on both measures. The mother was given an earpiece connected to a walky-talky so that a research assistant could provide instructions about the procedure without entering the room.

### The Caretaker Acute Stress Paradigm

The Caretaker Acute Stress Paradigm (CASP) was developed to observe the influence of maternal stress on maternal and infant reactivity within an experimental setting (59). The CASP has a standard 30-second physiology baseline and five episodes, each 2-min long. Mothers are seated in a standard chair, infants placed in a highchair facing the mother, close enough that the mother can touch and interact with her child. Following a resting baseline during which the mother sits quietly while the infant watches a video, the dyad engages in a face-to-face natural play (episode E1). The acute caretaker experimental episode (E2) follows in which the caretaker is exposed to an auditory and matching visual and auditory stressor or non-stress control condition. After a brief recorded introductory narrative that the infants were undergoing a medical procedure, caretakers in the experimental condition hear infant cries over headphones while watching matching images of crying infants on a screen in front of them. In the control condition, mothers hear infant vocalizations (e.g., cooing, gurgling) over the earphones while watching matched images on a screen and a recorded narrative that the infants were playing with an adult.

To ensure mothers focus on the experimental condition, and the infants would not be aware of the mothers' reaction, a screen was set up between her and her infant. All infants stayed in their highchair, were turned away, and a research assistant entertained them with bubbles and finger puppets for the 2-min of the stimulus episode. Thus, infants had the exact same experience regardless of maternal experimental condition.

**Figure 1:**
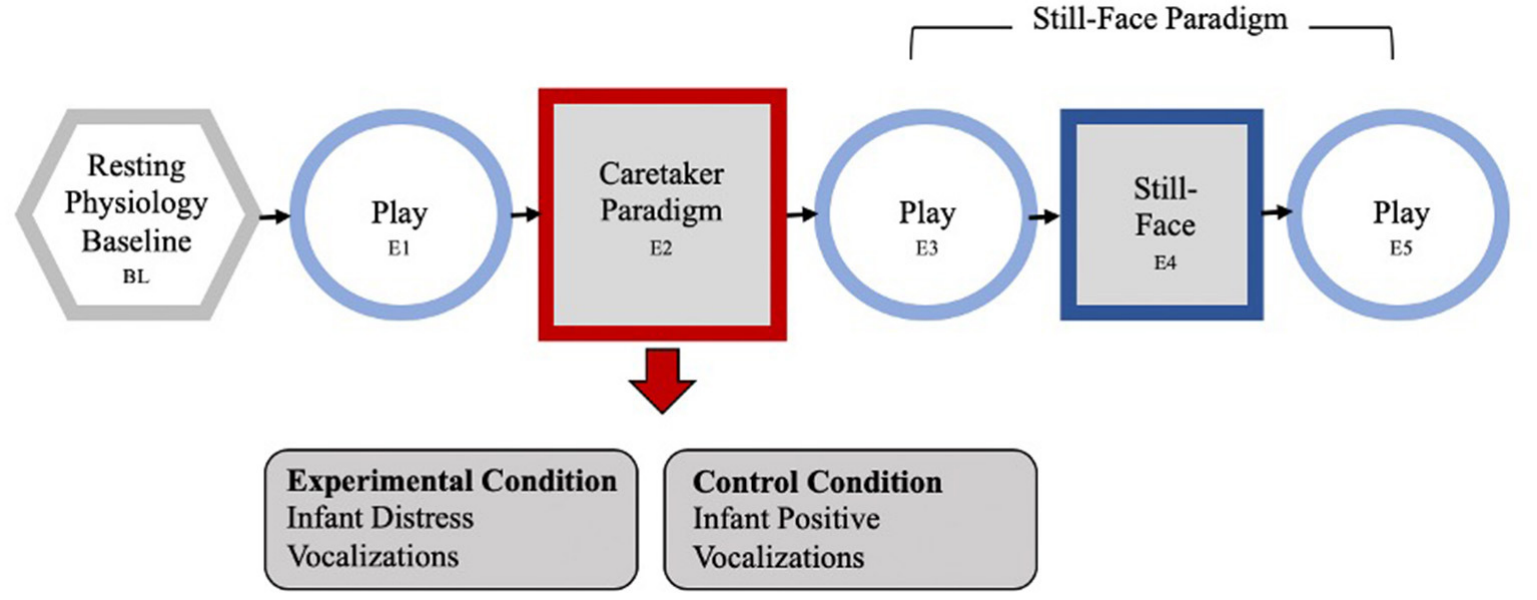
The Caretaker Acute Stress Paradigm, containing a standard 30-s physiology baseline and 5 episodes, each 2 min long. First the dyad engages in a face-to-face natural play (episode E1). The acute caretaker experimental episode (E2) follows: the caretaker is exposed to an auditory and matching visual stressor or control condition. Caretakers in the experimental condition hear infant cries, in the control condition mothers hear infant laughter and giggles over the earphones while watching matched images on a screen. Next, mother and infant were reunited for another face-to-face play episode (E3), followed by a classic still-face episode (E4) where the mother is asked to stop the interaction, sit back, and maintain a neutral “poker-face” (still-face). The final episode is another face-to-face play (E5).

Next, mother and infant were reunited for another face-to-face play episode (E3), followed by a classic still-face episode (E4) where the mother is asked to stop the interaction, sit back, and maintain a neutral “poker-face” (still-face). The final episode is another face-to-face play (E5; see Figure 1 for the entire paradigm). The CASP paradigm ended after E5 or was terminated early if an infant showed significant distress (e.g., crying) for more than 30 consecutive seconds. All infants who made it to episode E3 for at least 30 s were included in the study.

## Measures

### Maternal Depressive Symptoms

Maternal stress is often linked to maternal psychopathology, especially maternal depressive symptoms. To assess a possible impact of maternal depressive symptoms on the experimental manipulation, mothers were asked to complete the CESD-R (Center for Epidemiologic Studies Depression Scale-Revised) (65).

### Infant Stress Reactivity: Drop Out

The procedure was terminated if an infant showed distress for 30 consecutive seconds either in E3 (play episode after the caretaker stress), E4 (the still-face episode), or E5 (the reunion play episode after the still-face) and were labeled drop-outs. Drop-out episode (E3, E4, or E5) was recorded in the study notes and reviewed for accuracy (30 s distress) on the recorded video. The drop-out episode was then used as a measure of infant stress reactivity, to evaluate whether there was a difference in paradigm termination between the caretaker stress and control condition.

### Infant Stress Reactivity: Behavioral Distress

First sign of distress (e.g., the first negative vocalization or cry) was coded by two raters until an agreement was reached to compare if the caretaker-manipulation impacted the infants affect regulation, independent of the duration of that first sign of stress.

### Infant Heart Rate

Continuous cardiac data sampled at 1,000 Hz was collected on from mothers and their infants. Software from MindWare Technologies LTD was used for data cleaning and to generate the mean heart rate (HR) for mothers and their infants for each of the 2-min episodes of the CASP paradigm or the matched play-sessions of the control group.

### Statistical Analysis

All statistical analyses were carried out with IBM SPSS Statistics for Mac, version 23.

## Results

### Maternal Depressive Symptoms by Group

The average CESD score was 8.29 (*SD* = 5.654, range: 1–23) for the control group and 10.51 (*SD* = 8.895, range: 0–34) for the experimental group. General linear modeling showed that there was no significant difference in CESD score between groups (*p* = 0.141). In addition, there was no statistical difference in CESD scores between mothers of infants who dropped out of the paradigm due to too much distress compared to mothers of infants who did not drop out (*p* = 0.367).

### Infant Dropout After the Caretaker-Manipulation

**Figure 2:**
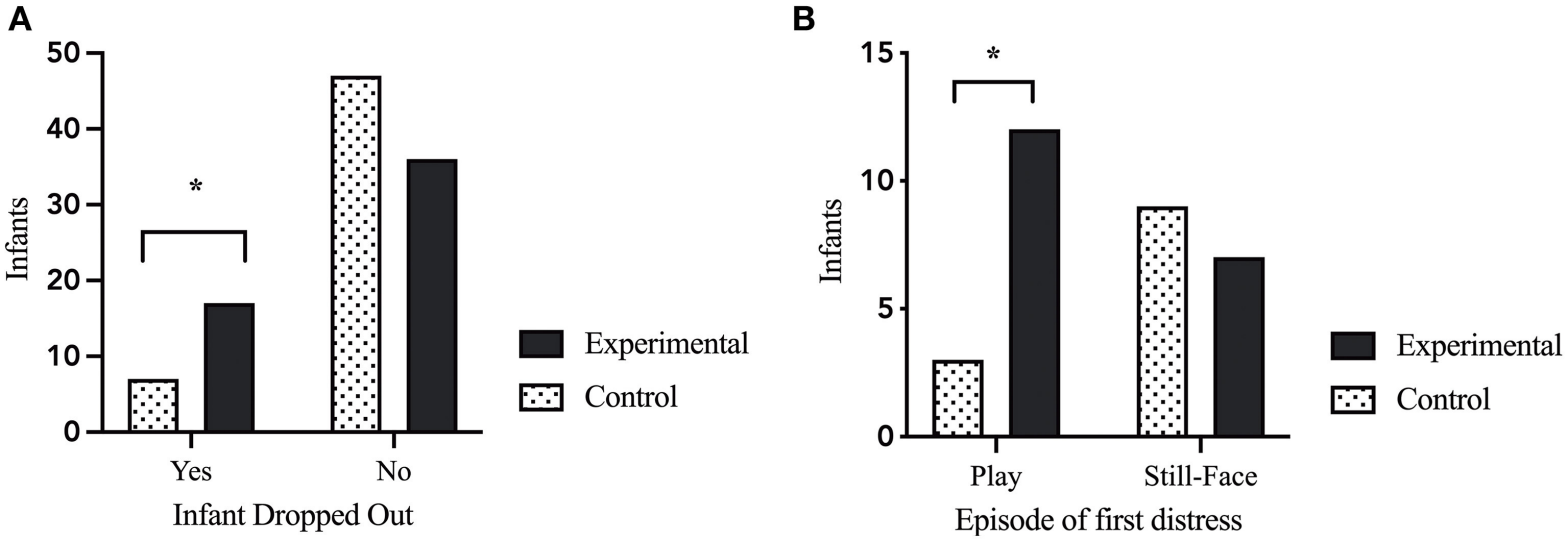
Infant behavioral stress reactivity. **(A)** Infant drop-out of the paradigm due to exhibition of distress crying for 30-consecutive-seconds. Infants in the maternal stress condition had to terminate the procedure significant more often than infants in the control condition [*X*^2^ = (1, *N* = 106) = 4.662, *p* = 0.031]. **(B)** Episode of first distress in the infants during the CASP paradigm. Infants in the maternal stress condition showed first distress significantly more often in the play episode after the caretaker-stress (or control) manipulation compared to infants of the maternal control condition [*X*^2^ = (1, *N* = 31) = 4.288, *p* = 0.038). *p* < 0.05*.

To evaluate the impact of the caretaker-manipulation on infant stress reactivity, we evaluated the number of infants who showed enough distress to terminate the paradigm. Chi-Square analysis comparing the caretaker-stress group with the control group revealed a significant difference in drop-out rate after the caretaker-manipulation. Significantly more infants in the maternal-stress group dropped out compared to infants in the maternal control group [*X*^2^ = (1, *N* = 106) = 4.662, *p* = 0.031; see Figure 2A].

### First Sign of Distress During the Paradigm

The episode of first distress was correlated with dropout episode [*r*_(105)_ = 0.699, *p* < 0.000]. A Chi-Square analysis showed that infants of mothers in the stress condition showed their first sign of distress during the (usually positive) play after the caretaker manipulation, while infants of the maternal control group had a higher rate of first distress during the following SF episode [*X*^2^ = (1, *N* = 31) = 4.288, *p* = 0.038; see Figure 2B].

### Differences in Infant Heart Rate

**Figure 3:**
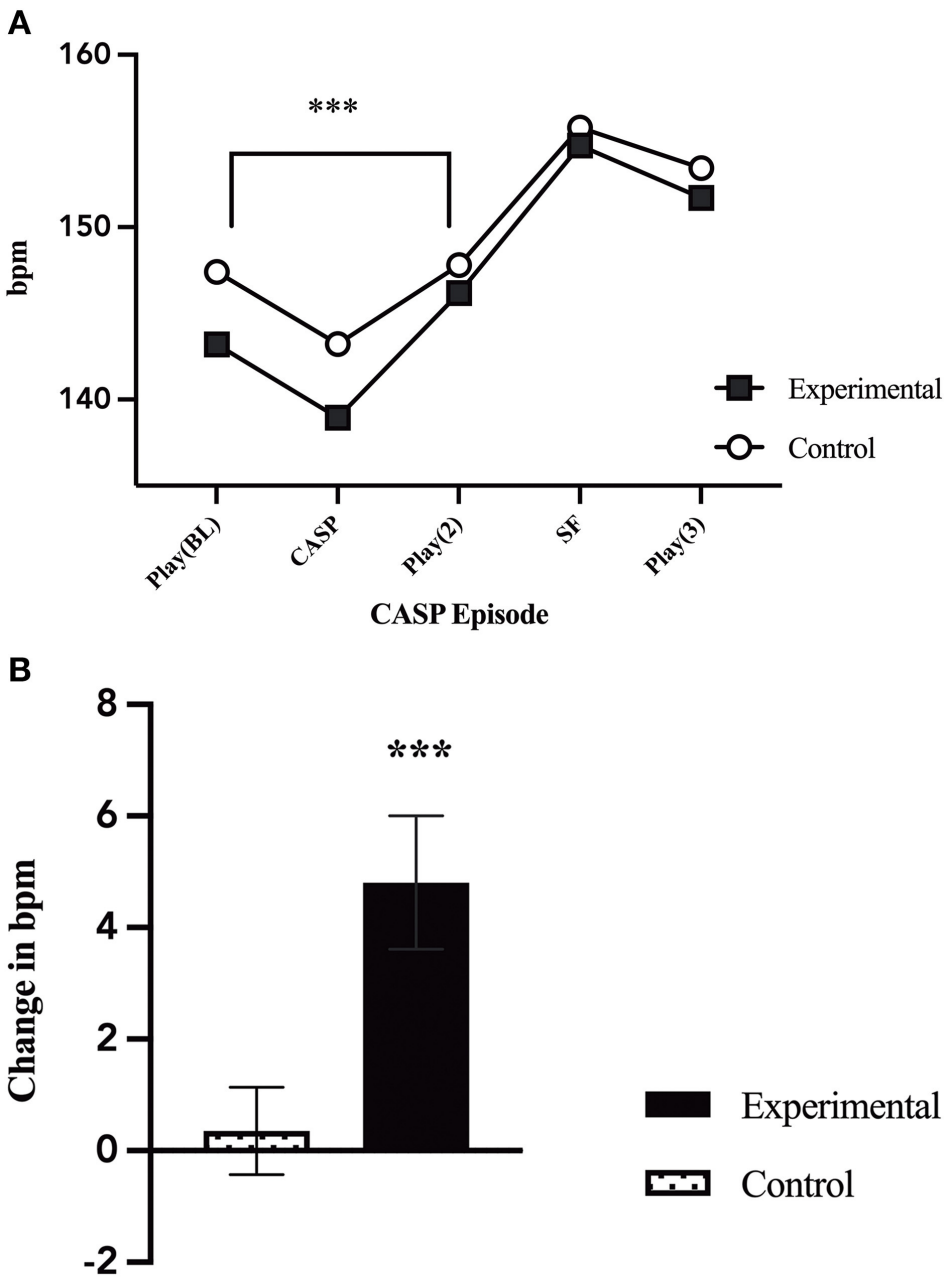
Changes in infant heart rate. **(A)** Infant average heart rate throughout the five CASP episodes by group. There was a significant increase in heart rate from play 1 (E1) to the second play (E3) for infant's who's mothers were in the caretaker-stress condition [*F*_(1, 102)_ = 9.81, *p* = 0.002]. **(B)** Comparison of the change in infant average heart rate during play 1 (E1), before caretaker manipulation, and play 2 (E3), after the caretaker manipulation, by group (caretaker-stress or caretaker control condition) [*F*_(1, 102)_ = 9.81, *p* = 0.002]. *p* < 0.01***.

Univariate general linear modeling showed that there was no difference between infant heart rate during baseline (*p* = 0.209). However, a repeated measure analysis comparing infant heart rate before and after the caretaker stress episode revealed a significant main effect of group by time [*F*_(1, 102)_ = 9.81, *p* = 0.002; Figure 3], with a significant increase in infant heart rate from the first play episode (E1) to the second play episode (E3), when mothers were in the stress condition.

### Differences in Infant Respiratory Sinus Arrhythmia

**Figure 4:**
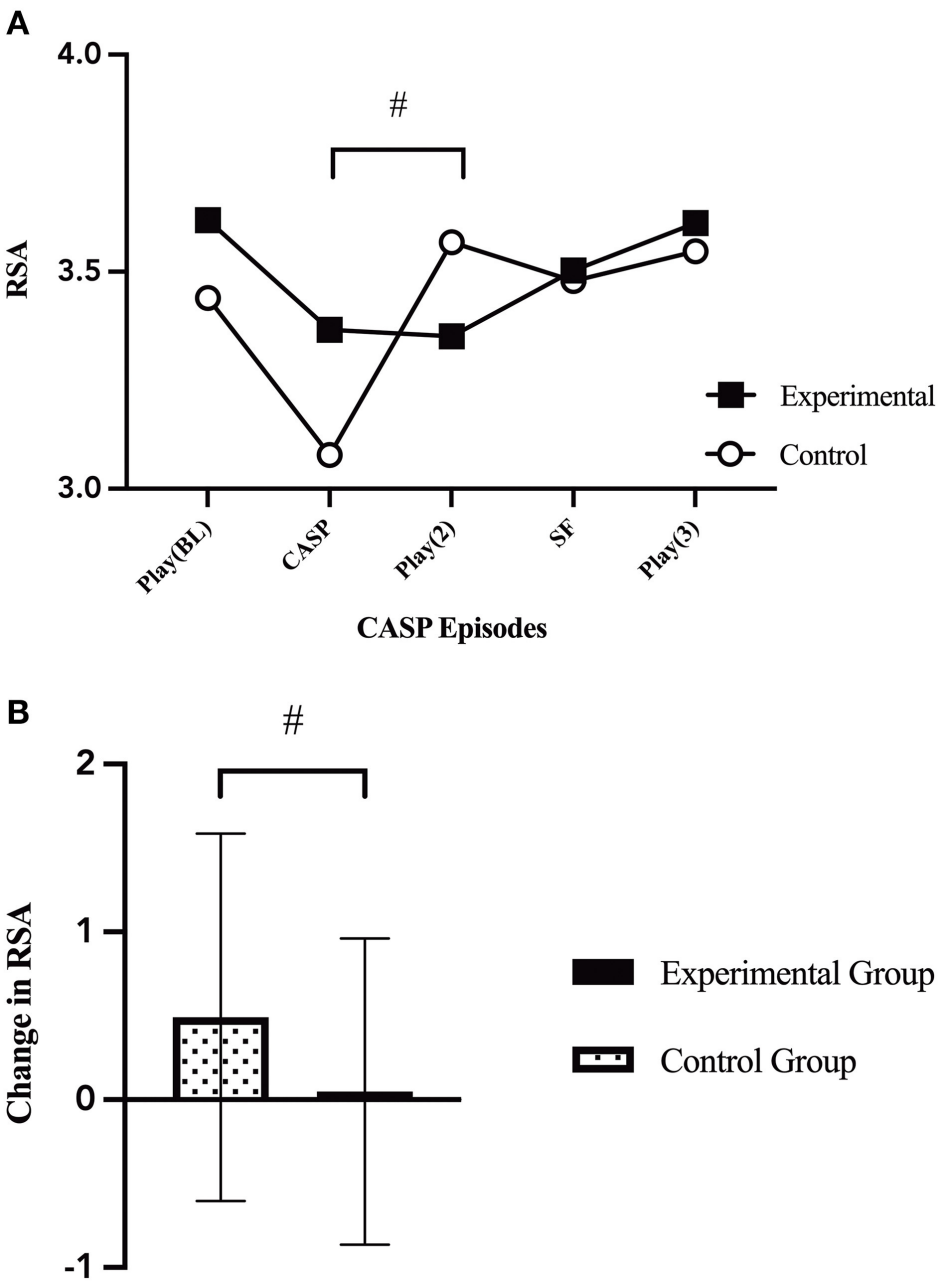
Changes in infant RSA. **(A)** Infant average RSA throughout the five CASP episodes by group [*F*_(1, 70)_ = 1.26, *p* = 0.294]. There was a marginal trend between grous in the change in infant RSA from the caretaker manipulation episode (E2) to the second play episode (E3) [*F*_(1, 75)_ = 3.539, *p* = 0.064]. **(B)** Change in infant average RSA from the caretaker manipulation (E2) to the second play episode (E3) [*F*_(1, 75)_ = 3.539, *p* = 0.064], by group (caretaker-stress or caretaker-control condition). *p* < 0.065^#^.

Univariate general linear modeling showed there was no significant difference in infant RSA by group at baseline (*p* = 0.386). Infants in the control condition showed a trend for faster RSA recovery [*F*_(1, 75)_ = 3.539, *p* = 0.064, Figure 4] during the second play episode (E3) after the caretaker control condition (E2) compared to infants whose mothers participated in the experimental condition (E2).

### Impact of Baseline Heart Rate on Infant Stress Reactivity

A regression analysis was performed to evaluate whether infant baseline heart rate may have contributed to the infants first sign of distress or drop out of the paradigm. Neither maternal nor infant baseline heart rate had a significant association with dropout episode (infant HR_BL_: *p* = 0.893; maternal HR_BL_: *p* = 0.124) or first episode to show distress (infant HR_BL_: *p* = 0.459; maternal HR_BL_: *p* = 0.346).

## Discussion

The present study explored the impact of maternal stress in a laboratory setting on subsequent infant regulation. The newly developed CASP paradigm (59) experimentally manipulates maternal stress in order to observe the immediate effects of maternal stress on the infant and dyad. Our results indicate that exposure to acute caretaker stress affects the infant and the dyad.

On a behavioral level, infants in the maternal stress condition compared to the infants in the control condition showed distress earlier in the paradigm, their distress was more intense, and more of them required early termination of the paradigm. First, infants in the caretaker-stress condition, showed first signs of distress on average more often during the play episode right after the caretaker-intervention, compared to infants whose mothers were in the control condition, who showed more often stress during the typical infant stressor (still face paradigm). The results indicate that maternal stress affects the infant not only during a dyadic challenge, but it has a disruptive effect even on the natural play after the caretaker-stress condition, which is typically expected to be a positive face-to-face play interaction with the mother. Second, infants in the caretaker-stress condition were more likely to require a termination of the experimental procedure as they showed more than 30-s of consecutive distress. It could be speculated that maternal stress depleted the dyadic resources to co-regulate over the full length of the paradigm. Interesting is also, that less infants of the caretaker-stress condition drop-out during the infant stress paradigm (still face). This could be explained with infants sensible to stress reaching their tolerable limit in the caretaker-stress condition earlier, not making it to the infant-stress episode. These experimental findings support the correlational findings by Pesonen et al. (66) that continued maternal stress over the pre- to postnatal period is associated with higher infant reactivity and by Feldman et al. (67) that infants of mothers with symptoms of depression showed less mature regulatory behaviors and more negative affect.

As regards physiology the infants in the experimental condition showed a significant increase in heart rate after the caretaker stress that was not observed in infants of mothers in the control condition. As the procedure was the same for all infants, the difference indicates that the acute stress in the experimental condition experienced by the caretaker was picked up, reacted to by the infant which may have interfered with the infants' self-regulation, as well as with the dyadic co-regulation. A similar trend was found for infant RSA, a measure of regulation rather than arousal. We observed that the infant RSA in both groups decreased, an indication of dysregulation during the caretaker stress (or control) condition, probably due to the brief separation from the mother. However, infants whose mothers were in the control condition had a faster RSA recovery as soon as the caretaker episode was over when they were reunited with their mothers. The majority of infants from the caretaker stress condition, contrariwise, did not show an RSA recovery after the infants were reunited with their mothers. The RSA recovery finding supports the original hypothesis of this study that caretaker stress leads to a disruption of regulatory resources, which then interferes with the dyad's ability to regulate stress (29, 30). Previous studies on maternal stress, namely maternal psychopathology, have found similar results, indicating a higher mean heart rate and weaker RSA recovery in exposed infants (38, 68).

Overall, the results indicate that even a brief and acute maternal stressor impacts infant physiology and emotional stress regulation. The findings are in line with research observing a lower dyadic ability to regulate after prolonged exposure to maternal stress (16, 34) and the observation that children exhibit an elevated stress response after exposure to high maternal stress (69, 70). However, most studies related to caregiver stress find that current levels and previous levels of high stress often overlap, making it difficult to distinguish whether previous or current exposure has a greater impact (70). The CASP paradigm allows us to investigate the impact of acute maternal stress on the dyadic interaction as well as the infants stress response. While acute stress may not be comparable to chronic exposure, it still allows for controlled laboratory observations with a control group to develop a better understanding how caregiver stress may affect the infants behavioral and physiological regulatory organization.

### Limitations and Future Directions

Limitations of this study include not accounting more detailed for maternal background. Previous studies have shown that maternal prenatal mental health, such as own adverse childhood experiences (ACE) or lifetime traumatic stress can affect infant regulation (69, 71). Future studies with the CASP should control for these measures or actively use them to create groups to learn more about the effect of maternal ACE or traumatic experiences on dyadic regulation of acute caretaker stress. The present study included current maternal depressive symptoms (CESD), while there was no statistical difference in maternal depressive symptoms between groups, there was a numerical difference in the highest CESD score in the caretaker-stress condition compared to the caretaker-control condition (34 vs. 23). However, we also tested if there was a difference in our main behavioral variable (infant drop-out of the paradigm) and found no statistical difference in maternal depressive symptoms of infants in the caretaker-stress and caretaker-control condition who had to terminate the procedure due to the 30-s consecutive distress limit.

Similarly, the majority of participating dyads were white. Previous research shows that maternal stress and factors that cause maternal stress, such as racism or lower socioeconomic status, are still more present in populations that belong to the global majority (back, indigenous, and people of color), which could have affected the results.

While the CASP is an attempt to measure maternal stress in a controlled laboratory setting, it measures acute, not chronic stress exposure. However, it would be important to investigate how these chronic factors (maternal exposure to racism, ACE, trauma, and SES) that have been shown to influence infant behavioral and physiological regulatory capacity affect the acute stress regulation in an experimental setting. This is one of the promising possibilities the new CASP paradigm opens research up to; it is possible to control for the effects of acute maternal stress, with a clear definition and allows to compare different maternal adversities, backgrounds, and preconditions. Large studies will allow cross-over designs, where dyads complete both the caretaker-stress and caretaker-control condition. Further, large studies could compare more than two groups, extending to maternal-healthy caretaker-stress, maternal-depression caretaker-stress compared to two control groups with matched diagnosis.

Remarkably, the findings indicate that even a brief laboratory stressor can affect the dyad in such a significant way that infant behavioral and cardiovascular regulation is compromised. Future research with the caretaker acute stress paradigm (CASP) will add to our understanding on the underlying mechanisms involved in the association between maternal depletion of resources and infant regulation. It would be especially interesting to observe dyads from a high-risk sample such as infants with depressed mothers. Results on the impact of maternal depressive symptoms have been equivocal but based on the literature one could assume a greater challenge of an additional caretaker stressor. However, it would be also within the scope of current literature to assume a better adaption of the infant to a stressed caretaker (for review, see (72)) and subsequently a better adjustment of the dyad to the CASP.

Overall, the CASP offers new opportunities to study the independent effects of maternal stress on the dyad and its interplay with other risk factors. Observations within a controlled laboratory setting will allow us to gain new insight into subtle variations of caregiving under stress in a more objective way than previous studies, extending our understanding of the underlying behavioral and physiological mechanisms associated with maternal stress.

## Data Availability Statement

The raw data supporting the conclusions of this article will be made available by the authors, without undue reservation.

## Ethics Statement

The studies involving human participants were reviewed and approved by IRB office at the University of Massachusetts Boston. Written informed consent to participate in this study was provided by the participants' legal guardian/next of kin.

## Author Contributions

IM, NS, JD, and ET contributed to conception and design of the study. JD and IM organized the database. IM performed the statistical analysis and wrote the first draft of the manuscript. All authors contributed to the article and approved the submitted version.

## Funding

This research was supported by grants from the National Science Foundation 1457111 (ET and NS), NICHD R01 HD083267-01 (ET), The Bial Foundation (ET), and NICHD R03 HD095818 (ET).

## Conflict of Interest

The authors declare that the research was conducted in the absence of any commercial or financial relationships that could be construed as a potential conflict of interest.

## Publisher's Note

All claims expressed in this article are solely those of the authors and do not necessarily represent those of their affiliated organizations, or those of the publisher, the editors and the reviewers. Any product that may be evaluated in this article, or claim that may be made by its manufacturer, is not guaranteed or endorsed by the publisher.
